# Indicators for a circular economy in a regional context: an approach based on Wielkopolska region, Poland

**DOI:** 10.1007/s00267-023-01887-w

**Published:** 2023-09-26

**Authors:** Justyna Cader, Renata Koneczna, Artur Marciniak

**Affiliations:** 1 Mineral and Energy Economy Research Institute, Polish Academy of Sciences, ul. Wybickiego 7A, 31-261 Kraków, Poland; 2 Institute of Geophysics, Polish Academy of Sciences, ul. Księcia Janusza 64, 01-452 Warszawa, Poland

**Keywords:** Circular economy, Regional monitoring, Indicators, Strategy, Wielkopolska region

## Abstract

In recent years, the European Commission has made a significant commitment to transition to a circular economy (CE). At the same time, tracking progress in CE implementation remains a major challenge, especially at the regional level. In this context, a set of CE indicators has been proposed for key areas of a selected region of Poland – Wielkopolska, as an area promoting a holistic approach to development through the CE concept. The available scientific literature and key national and regional policies were reviewed. In addition, a desk-research analysis of 22 CE strategic documents of European regions was performed. Applying the aforementioned methods and expert interviews, a set of key 93 indicators was selected within the province’s dominant industries, such as agri-food, industrial processing, mobility and transport, construction and energy. Also, focus was paid to the socio-innovation area. The proposed framework for tracking CE development allows adequate capture of CE’s effects at the regional level. They also provide recommendations for creating monitoring in regions with similar economic profiles.

## Introduction

The idea of sustainable development is based on a simple premise - the needs of the current generation can and should be met without diminishing the chances of future generations (United Nations 1987). A medium that can be used by various countries and individual regions to achieve sustainable development is the circular economy (CE) (Rodriguez-Anton et al. 2019). In contrast to the linear economy, CE aims to minimize or eliminate inputs from fossil or non-renewable sources in the production system and maximize the reuse of the materials (Korhonen et al. 2018). This approach covers sharing, leasing, reusing, repairing, refurbishment and recycling existing materials and products to extend their life cycle (European Parliament 2022).

In recent years, the European Union (EU) has taken particularly intensive steps toward a transformation to a circular economy. CE is a priority of the EU’s economic policy for the coming years and one of the key drivers for the implementation of the principles of responsible, sustainable development (EC 2020a), while contributing to the achievement of the Sustainable Development Goals (United Nations 2023). Simultaneously, Europe can be considered the most innovative environment for the development of the circular economy on a regional scale (Gravagnuolo et al. 2019).

The transition to a CE has been indicated by the EU in several key documents, including two action plans. The first indicates that the value of products, materials and resources in the economy should be maintained for as long as possible, and waste generation minimized (EC 2015). The second action plan addresses accelerating the transition to a regenerative growth model, reducing the consumption footprint and increasing the use of recycled materials (EC 2020a). In addition to the action plans, the European Commission (EC) recommended the development of CE strategy documents initially at the national level, and later also at the regional, local and sectoral levels.

Currently, according to the European Circular Economy Stakeholder Platform (ECESP), 64 strategies for the transition to a CE have been implemented in EU member states, including 22 regional strategy documents (strategies, action plans, agendas, roadmaps) (EESC 2017). These documents are differentiated due to the specifics of the region, including their socio-economic conditions. They identify sectors, requiring CE implementation.

However, monitoring CE remains a major worldwide challenge. A diverse set of CE metrics can be observed (Parchomenko et al. 2019). There is a noticeable lack of one specific set of indicators or an acceptable synthetic indicator for monitoring CE implied at different levels and in many countries (EEA 2016). The wide variety of indicators may also be due to the varying attitudes of stakeholders towards the CE concept, which makes the process of CE monitoring more challenging (Mazur-Wierzbicka 2021).

A set of indicators at the macro level has been implemented in EU Member States (EC 2022). Nonetheless, European regions are still struggling with building specific monitoring and evaluation measures for CE (Vanhamäki et al. 2021). Analyses of indicators and approaches to monitoring CE at the global or national level, and the possibility of adapting such approaches to European regions, have shown that existing tools are insufficient (Avdiushchenko and Zając 2019).

The regional focus is a relatively neglected dimension in terms of theoretical contributions to CE performance evaluation, but the transition to a circular economy requires region-specific policy design (Madden et al. 2019; Hildebrandt et al. 2020; Silvestri et al. 2020). In most regions, monitoring and assessment of the realization of CE strategies and agendas are in the early stages of development, indicating that regions have different approaches to these actions (Vanhamäki et al. 2020). The differences in opportunities for achieving a circular economy in various areas of a country can be significant. This is due, among other things, to differing demographics, economic conditions, and cultural backgrounds (Ning 2012). CE monitoring provided at sub-national levels will enable regions with poor performance to be supported more effectively, while at the same time, it is possible to employ practices of the most prosperous regions (Heshmati and Rashidghalam 2021). Muizniece et al. (2019) have proposed a bottom-up approach to implementing the concepts of bioeconomy and circular economy taking into account the specific capabilities and resources of small regions, rejecting the use of generalized assumptions at the national level. Kristensen and Mosgaard (2020) also emphasized the importance of designing sector-specific indicators to increase the efficiency of CE deployment through the concretization of activities.

Monitoring indicators should reflect the socio-economic and technological conditions of the regions, including their specializations, regional strengths and potential (Avdiushchenko and Zając 2019; Vanhamäki et al. 2020). Considering the importance and complexity of the CE concept, it is essential to analyze the situation in the region, identify the basic conditions of its development, and further actions towards CE, which will allow constructing recommendations for comprehensive support of the transition to this economic model. Implementation of monitoring indicators at the regional level enables more direct feedback (Hildebrandt et al. 2020).

To address this research subject, we proposed how to select a CE monitoring framework for a chosen European region. In addition, we emphasized the importance of developing indicators dedicated to specific sectors, which would allow increasing the efficiency of CE implementation through the concretization of activities. For this purpose, an analysis of the provisions in regional strategic documents describing the priority directions of the region’s development was used. An extensive review of the EU regional strategies for the circular economy and the scientific literature was also conducted, focusing on identifying CE implementation indicators. These activities, combined with expert interviews, resulted in the selection of relevant indicators within the region’s sectors.

The study was conducted as a case study for Poland’s second-largest voivodeship – Wielkopolska. None of the 16 existing Polish regions has an action plan or CE strategy. However, Wielkopolska is currently the only voivodeship in the country with an established CE regional support concept, which is a contribution to the creation of an official CE roadmap of this region (Wdowin et al. 2021). Furthermore, Wielkopolska was one of five EU member regions involved in the CIRCE2020 project to develop and test business models that steer industrial sectors in a more sustainable direction. One of the horizontal goals of the province’s strategy is the sustainable development of the region, including zero-emissions, electromobility, green energy, energy transformation, decarbonization, and circular economy (Board of the Wielkopolska Region 2020a).

Consequently, the proposed research results should facilitate the transition to the CE model in the analyzed region. The article contributes to the selection of relevant indicators for the target region, while encouraging other provinces to develop customized systems for tracking CE implementation. It will also provide recommendations for the selection of effective metrics that could constitute a benchmark for a CE monitoring framework in regions with similar economic profiles. Simultaneously, there is no contraindication to the implementation of a selected group of indices by other regions according to preference, for instance, metrics from the socio-innovation area, or as a basis for the development of final and complex CE indicators in regional policy.

## Methods and data

**Fig. 1:**
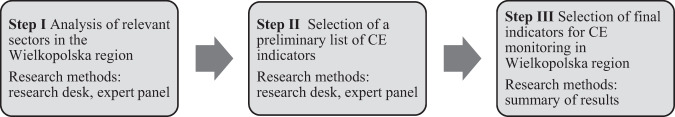
Diagram of the research procedure

The study was carried out using an analysis of available source materials, including strategic and planning documents operating at European, national and regional levels, as well as individual regional strategies of EU members. The scientific literature on CE issues was also used, along with expert analysis. The study was divided into three key stages (Fig. 1).

### Desk research

The comprehensive review included the available scientific literature, as well as strategy and planning documents at national and regional levels listed in “Poland and Wielkopolska CE monitoring”. An important source of data was the ECESP platform, with regional strategies of EU members (EU regional CE monitoring) as well as a developed set of CE indicator frameworks at the national level in EU countries (European Commission 2020a, 2022).

The first step revealed the dominant sectors for Wielkopolska. They were determined using data published in key strategies for Wielkopolska.

In the next step, indicators were selected concerning sectors based both on the mentioned legal acts for Wielkopolska, and national strategies and by analyzing materials developed at the regional level on the ECESP platform. The article considers the region at NUTS 2 and NUTS 3 levels (Statistical Office of the European Communities 2022). The region as an administrative unit is crucial in the context of EU development policy and represents the optimal level of implementation for functional CE (Arsova et al. 2021).

**Fig. 2:**
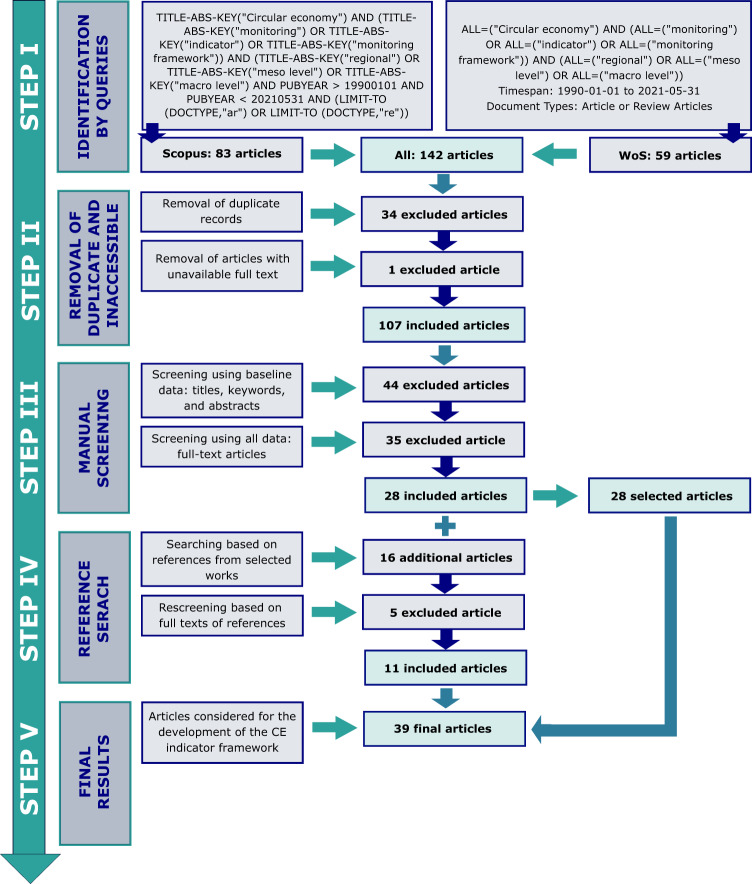
Scheme of selection of scientific articles in the field of CE indexing

Additionally, an analysis of research papers in the field of CE indexing at the regional level has been performed. The analysis used scientific papers (original research and review papers) collected in Scopus and Web of Science (WoS) databases. The screening included papers published from 01-01-1990 to 05-31-2021 (before the scheduled expert interviews). The selection of articles from the scientific database was carried out based on identified keywords and their combinations, which are summarized in Fig. 2.

Selected articles served as supporting literature for the choice of individual indicators. The specific links are highlighted in Table A in the Supplementary material.

Most of the considerations for CE indices at the regional level concern Chinese cases (Saidani et al. 2019). China’s CE evaluation system mainly includes indicators for resource output, resource consumption, integrated resource utilization, waste disposal, and pollutant emission (Geng et al. 2012; Hu et al. 2018; Ye et al. 2022). Focus at the macro-level is strongly related with material flows and their circularity (Gao et al. 2020; Harris et al. 2021; Virtanen et al. 2019). Tang et al. (Tang et al. 2020) focused on developing a monitoring system for Chinese provinces by combining the characteristics of the industrial CE from the perspective of material flow analysis (MFA) and Chinese index systems. Another index system for the selected province was proposed by Qing et al. (Qing et al. 2011), which included five aspects (social and economic development, resource efficiency, recycling and reuse of resources, environmental protection, pollution reduction). Geng et al. (2009) compared sub-provincial city Dalian with other city-provinces within the resource consumption, waste discharge, waste treatment, and waste reclamation areas. However, several individual indices were selected for Dalian municipality related to its own industrial advantages and bottlenecks.

In the case of European regions, the promotion of CE as a strategic priority in the development of regional specializations made it possible to define CE goals and activities focusing on existing local assets, innovation and future potential (Vanhamäki et al. 2021). Avdiushchenko and Zając (Avdiushchenko and Zajaç 2019) noted that existing CE monitoring in Europe does not sufficiently cover changes in consumption and production patterns, the spatial dimension affected by CE strategies, and the social, economic and cultural transformation caused by the reorientation to CE-based regional development. Hence, they proposed a set of indicators to provide an additional instrument to support the various monitoring activities aimed at tracking the effects of CE adaptation in the European context. Eco-innovation is also inextricably linked to CE issues at the regional level (Drejerska et al. 2020; Smol et al. 2017). Smol et al. (Smol et al. 2017) recommended using five groups of indicators to measure CE-eco-innovation, i.e., eco-innovation inputs, activities and outputs, as well as effects of introducing eco-innovation: environmental resource efficiency and socioeconomic outcomes. In addition, the importance of creating guidelines for monitoring processes at the regional level through smart specializations was presented by Stanojev and Gustafsson (Stanojev and Gustafsson 2021).

A mult–criteria assessment of the potential for the development of a closed-loop economy at county level was proposed by Strat et al. (Strat et al. 2018). Based on a comprehensive six-dimensional index built on 16 individual variables, the potential for CE in Romanian regions was described. Heshmati and Rashidghalam (Heshmati and Rashidghalam 2021) also proposed a multidimensional indicator of eight elements for analyzing CE development at the level of Swedish municipalities. The analysis shows that individual regional units should apply measures matched individually to their economic and resource characteristics. Another approach is to use the level of adoption of different CE-related activities. Scarpellini et al. (Scarpellini et al. 2019) used for this purpose indicators such as employment, turnover and volume of raw material consumption in the Aragon region. For the same Spanish region, Aranda-Usón et al. (Aranda-Usón et al. 2020) determined the gradual adoption of the circular economy by the regional business sector. In their analysis, they used the share of companies that implement CE-measures related to recycling and energy efficiency, dematerialization, renewable energy, recycled raw materials, industrial symbiosis solutions, collaborative circular practices, eco-innovation and eco-design, taking into account the average turnover of these companies. The inclusion of the business sector in monitoring CE implementation is relevant, as companies are highly sensitive to the existence of favorable regional conditions (Aranda-Usón et al. 2018).

Monitoring systems at the regional level are often based on CE final targets and the 3 R/4 R (reduce, reuse, recycle/and recover) principles (Banaite and Tamošiuniene 2016; Geng et al. 2012; Qing et al. 2011). Some researchers use a system of indicators based on ecological efficiency theory (Huang 2015; Liu et al. 2018; Wang et al. 2015).

### Expert panel

The expert panel was applied in both Step I and Step II of the research to select the relevant sectors/areas for the Wielkopolska region in the context of CE, and then to identify the CE indicators essential for monitoring the implementation of this model.

We assembled a panel of purposefully engaged experts from different sectors and fields (Table 1) to capture and integrate multiple actors and disciplines and hence perspectives, required to achieve a holistic view of CE (Murray et al. 2015). The panel comprised experts with expertize in different CE concepts from academia, business, partnerships/cooperatives, and non-governmental organization. This diverse panel enabled the exchange between theory and practice (Chan et al. 2015).

**Table 1:** The outline of the experts involved in the study

No.	Position	Organization	Type
1.	Professor, Head of the Department of Industrial Products and Packaging Quality	University	Academia
2.	Vice-Dean for Development, Program Leader in the field of CE	University	Academia
3.	Head of the Scientific Center (in the field of engineering of anthropogenic minerals)	University	Academia
4.	Professor, Head of the Ecotechnology Laboratory	University	Academia
5.	Researcher, Head of the Laboratory of Biogenic Raw Materials	Research institute	Academia
6.	R&D Manager in the Innovation and Technology Section	Small and medium-sized enterprises	Business
7.	Managing Director in environmental protection areas, waste and packaging management	Small and medium-sized enterprises	Business
8.	Development Director in environmental protection areas, waste and packaging management	Small and medium-sized enterprises	Business
9.	Managing Director	Cluster coordinator; not-for-profit	Business; Partnerships/Cooperative
10.	Managing Director	Cluster coordinator; not-for-profit	Business; partnerships/cooperative
11.	Coordinator of the department for CE, Vice-President of the biorecycling association	Think-tank, association	Non-governmental organization; Partnership/Cooperative
12.	Specialist in environmental protection and sustainable development	Foundation	Non-governmental organization

In the case of Academia experts, representatives from Wielkopolska scientific institutions and representatives of higher education were selected. In particular, the expert’s experience was considered, i.e., length of scientific seniority, scientific activity expressed through the type and number of thematic publications, functions held in institutions, and involvement in scientific projects at the national and international level related to CE.

For the remaining groups Business, Partnerships/cooperative, and Non-governmental organization experts were selected through membership in dedicated associations and clusters of waste management, recycling, eco-management, clean production, as well as conference speeches or participation in working/project groups related to the development of a sustainable, low-carbon, resource-efficient and competitive economy. Individual profiles were also analyzed using international social networks. Three representatives assigned exclusively to the Business group stood out for their activity in the areas of sustainability and CE-related innovation.

Individual invitations were sent to the selected group of experts via email. Subsequently, they were contacted by phone acquainting specialists with the objectives of the study and its process. Experts received an interview script before direct interviews. Telephone or Online In-Depth Interviews were conducted sequentially. In-depth interviews were selected because of the possibility to obtain sufficiently comprehensive answers and additional explanations for the questions contained in the interview scenario. The interviews were conducted in 2021. The length of the interviews was not limited by time, while individual interviews lasted from 50 min to 1.5 h.

The expert interview was divided into two main parts: 1. Identification of key sectors in Wielkopolska and 2. Indicators for assessing the degree of development of CE (Table 2). Questions in the first part enabled the identification of key sectors in Wielkopolska. In the second part, respondents highlighted the most relevant CE indicators matched to the sectors.

**Table 2:** Scenario of interviews with experts

Interview parts	General topic	Exemplary questions
Part 1	Identification of key sectors in Wielkopolska	Can you identify effectively developing industries in the region? Can you provide examples of the most effective local activities/good practices in selected areas and industries within CE?
Part 2	Indicators for assessing the degree of development of CE	Could you indicate the best indicators for the assessment of CE implementation in the region?

## Results

### Identification of dominant sectors

Based on the proposed research methodology, five main sectors and one area of intervention additionally highlighted by experts were selected. These sectors are:

- Agri-food,
- Construction,
- Industrial processing,
- Mobility/Transport,
- Energy,

And Socio-innovation area.

The agri-food sector is engaged in the production of food products and intermediates for consumption. Industrial processing, on the other hand, is defined as the physical or chemical processing of raw materials, materials or intermediate products into a new product. The third sector analyzed is construction, which includes general construction and specialized works involving the construction, reconstruction, as well as repair and demolition of buildings. The transport and mobility sector is defined as activities related to the transport of people or goods using various modes of transport and supporting infrastructure. The fuel and energy sector deals with the extraction of energy resources and their processing and distribution of the energy produced.

In addition, a socio-innovation area was distinguished as a necessary component of CE implementation. In the CE concept, there is a broad spectrum of issues such as education, green procurement, sharing economy, and CE initiatives, which, according to expert indications, are vital for monitoring the proper execution of the CE model. Innovation and social issues are directly related to CE (Smol et al. 2017; Avdiushchenko 2018).

### EU regional CE monitoring

In the CE monitoring framework established by the European Commission, there are no regional indicators, but only those at the national level. They cover value chains like production and consumption, waste management, secondary raw materials and competitiveness and innovation. There are 23 assigned indicators listed (EC 2018).

The monitoring of CE by European regions is partly in line with EC monitoring at the national level. Regional indicators have been included in regional CE strategies, action plans, agendas, and roadmaps (EESC 2017). These are comprehensive documents that consider the CE as an overall concept for regional development.

Most of the regions’ strategies have defined indicators that relate to the specializations of these areas (Table 3). There are a total of 714 indicators in all the regions under discussion, which are sometimes similar or identical. The largest number of CE monitoring indicators is presented in the Spanish regions. Significantly, the main CE areas of the analyzed regions fit into the specializations of Wielkopolska’s sectors. All regions indicate the importance of the socio-innovation area.

**Table 3:** Sectors/area in CE regional strategy papers in the EU

No.	Country	Region	Year of publication	Sector					Area	Number of indicators	Citation
				Agri-food	Construction	Industrial processing	Mobility/ transport	Energy	Socio-innovation		
1	Austria	Große Walsertal (covers Vorarlberg region)	2020	x	x	x	x	x	x	9	Regio Großes Walsertal 2020
2	Belgium	Brussels-Capital Region	2016		x		x	x	x	15	Government of the Brussels-Capital Region 2016
3	Belgium	Flanders	2020	x	x	x	x	x	x	89	Circular Economy Policy Research Center 2020
4	Belgium	Wallonia	2021	x	x	x			x	80	Walloon Public Service 2021
5	Finland	Ostrobothnia	2020			x			x	-	Jakobstad Region Development Company Concordia 2020
6	Finland	Central Finland	2018		x		x		x	16	Circwaste 2018a
7	Finland	North Karelia	2018	x	x	x	x		x	16	Circwaste 2018b
8	Finland	South Karelia	2018		x	x	x		x	16	Circwaste 2018c
9	Finland	Southwest Finland	2019		x		x		x	16	Circwaste – Materiaalit kiertoon 2019
10	Finland	Päijät-Häme	2021	x	x	x	x	x	x	19	The Regional Council of Päijät-Häme 2021
11	Italy	Emilia-Romagna	2019	x		x			x	2	Regione Emilia-Romagna 2019
12	Netherlands	Chemport (covers: the Province of Groningen, the Province of Drenthe)	2020	x		x		x	x	-	Chemport Europe 2020
13	Netherlands	Friesland	2021	x	x				x	1	Circulair Friesland 2021
14	Portugal	Madeira	2021	x	x				x	10	Portuguese Regional Directorate for the Environment and Climate Change 2021
15	Portugal	Tâmega e Sousa	2019	x	x	x	x	x	x	-	Intermunicipal Community of Tâmega and Sousa 2019
16	Spain	Andalusia	2018	x				x	x	144	Ministry of Agriculture Fisheries and Rural Development 2018
17	Spain	Aragón	2020	x		x	x		x	8	Government of Aragón 2020
18	Spain	Castilla-La Mancha	2021	x	x	x			x	29	Dirección General de Economía Circular CLM 2021
19	Spain	Catalonia	2015	x		x		x	x	81	Government of Catalonia 2015
20	Spain	Extremadura	2017	x	x	x		x	x	48	Regional Government of Extremadura 2017
21	Spain	Galicia	2021	x	x	x	x	x	x	106	Xunta de Galicia 2021
22	UK	Scotland	2016	x	x			x	x	9	The Scottish Government 2016

### Poland and Wielkopolska CE monitoring

In Poland, the document strictly dedicated to CE is the roadmap, which was adopted by the Council of Ministers in 2019 (The Council of Ministers 2019). The map is in line with the European Commission’s goals for the transition to a closed economy in terms of four courses of action: sustainable industrial production, sustainable consumption, bioeconomy, and new business models. In addition, the act refers to the need to develop indices of the impact of CE at the level of regions and the national economy, but such indicators have not been developed.

At the same time, the authorities created various strategic documents and action frameworks that have relevant synergies with the Polish CE roadmap (Table 4). However, these documents are general in scope, and it is difficult to identify a set of indicators for monitoring CE, even in regional acts.

**Table 4:** CE-related indicators in national and regional documents

No.	Title	Document type	Year of publication	Number of indicators	Citation
1.	National Waste Management Plan 2022	national	2016	119	The Council of Ministers 2016
2.	The Strategy for Responsible Development for the period up to 2020 (including the perspective up to 2030)	national	2017	72	The Council of Ministers 2017
3.	National Strategy of Regional Development 2030	national	2019	21	The Council of Ministers 2019
4.	The National Environmental Policy 2030	national	2019	20	The Ministry of Environment 2019
5.	National Energy and Climate Plan for the years 2021–2030	national	2019	-	Ministry of State Assets 2019
6.	Road Map towards the Transition to Circular Economy	national	2019	-	The Council of Ministers 2019
7.	Development strategy of the Wielkopolska Region until 2030	regional	2020	11	Board of the Wielkopolska Region 2020a
8.	Regional Innovation Strategy for Wielkopolska 2030	regional	2020	69	Marshal’s Office of the Wielkopolskie Voivodeship 2020
9.	Environmental Protection Program for the Wielkopolska Region until 2030	regional	2020	106	Siudak et al. 2020
10.	Waste Management Plan for the Wielkopolska Region for 2019–2025 with an Investment Plan	regional	2020	52	Board of the Wielkopolska Region 2020b
11.	Wielkopolska Regional Action Plan for Sustainable Energy and Climate in the field of renewable sources and energy efficiency with a perspective until 2050	regional	2021	-	Board of the Wielkopolska Region 2021

### Proposal of CE monitoring for Wielkopolska

Analysis of documents dedicated to CE and a panel of experts allowed selecting indicators that fit into CE and linking them to key sectors of the Wielkopolska region. Some of the indicators are not published in available databases or statistical yearbooks of the province. They require to be implemented in the reporting of public administration and enterprises. Totally, 93 indicators were selected, including for each group: agri-food – 20, construction – 12, industrial processing – 21, mobility/transport – 13, energy – 16, and in the area of socio-innovation – 11 (Table 5). The proposed indicators or their scopes can be found in EU regional strategies or scientific articles. At the same time, each of the presented indicators was recommended in the expert panel. Detailed information on the links of indicators to regional strategies and scientific articles is provided in Table A in the Supplementary material.

**Table 5:** Selected indicators by sectors and area for the Wielkopolska region

Sector/area	Indicators	Unit
AGRI-FOOD SECTOR	The amount of food waste	Mg
	Share of food waste from the sector to total food waste	%
	Share of managed waste generated in the sector to total waste	%
	Plastic usage	Mg
	The amount of managed livestock by-products	Mg
	The amount of biomass product per unit area	Mg/ha
	Total consumption of synthetic N and P fertilizers in agricultural production per unit area	Mg/ha
	Total consumption of organic N and P fertilizers in agricultural production per unit area	Mg/ha
	Percentage of land with maintained or improved soil quality relative to total land	%
	CO_2_ emissions in relation to the CO_2_ total emissions	%
	Nitrogen compound emissions (NH_3_, NO_x_) to the nitrogen compound total emissions	%
	Water consumption	m^3^
	Share of the agri-food sector in total water consumption	%
	Share of reused water in total water use	%
	The amount of rainwater used	m^3^
	Number of producers with organic certification	number
	Number of organic farms per unit area of agricultural land	number/1000 ha
	Percentage of the cultivated area under organic production	%
	Energy consumption of the sector in relation to total demand in the region	%
	Number of agro-ecological initiatives	number
CONSTRUCTION SECTOR	Share of construction and demolition waste in general waste	%
	The amount of construction and demolition waste generated	Mg
	Share of managed construction and demolition waste in the total amount of waste	%
	Recycling rate of construction and demolition waste	%
	Products and construction techniques covered by life cycle analysis studies	number
	Construction works with circular design	%
	Share of reused excavated soil in the total amount of construction and demolition waste	%
	Share of new zero-emission buildings in the total number of new buildings	%
	Share of RES in total energy consumption in public buildings	%
	Share of public buildings requiring thermal modernization	%
	Construction works with a minimum level of materials reuse (%)	%
	Share of buildings with certification in relation to all buildings	%
INDUSTRIAL PROCESSING SECTOR	Industrial value added in the key sectors*	Euro
	Total amount of generated industrial waste	Mg
	Industrial waste generation per unit of industrial value added	Mg/Euro
	Industrial waste generation in the key industrial sectors*	Mg
	Recycling rate of industrial solid waste	%
	Industrial waste produced as % of the total waste produced	%
	Industrial waste reused as a source of raw materials in relation to total waste	%
	CO_2_ emissions resulting from production processes	Mg
	SO_2_ emissions resulting from production processes	Mg
	Share of the sector in GHG emissions	%
	Energy consumption per unit of industrial value added	ktoe/TEUR
	Energy consumption per unit of production in the key industrial sectors*	ktoe/TEUR
	Energy consumption of the key industrial sectors in the total regional energy consumption	%
	Water intensity of industry – industrial water consumption per unit of industrial value added	m^3^/TEUR
	Water consumption per unit of production in the key industrial sectors*	m^3^/TEUR
	Industrial wastewater generation per unit of industrial value added	m^3^/TEUR
	Reuse rate of industrial water	%
	Life cycle assessment of enterprises activity (amount companies with LCA reports)	number
	Number of EMAS implementations in entities	number
	Number of Ecolabel-certified products/services	numbers
	Industrial and territorial ecology projects	numbers
MOBILITY AND TRANSPORT SECTOR	Passenger transport – total movement of passengers using public transport	pkm
	Share of petrol-powered vehicles to total registered vehicles, with their number for:	%
	– passenger vehicles, mass passenger transport vehicles, freight transport vehicles	number for each type
	Share of diesel-powered vehicles to total registered vehicles, with their number for:	%
	– passenger vehicles, mass passenger transport vehicles, freight transport vehicles	number for each type
	Share of electric vehicles to total registered vehicles, with their number for:	%
	– passenger vehicles, mass passenger transport vehicles, freight transport vehicles	number for each type
	Share of hydrogen vehicles to total registered vehicles, with their number for:	%
	– passenger vehicles, mass passenger transport vehicles, freight transport vehicles	number for each type
	Environmental cars in the municipal organization	number
	Number of stations and charging points for electric vehicles	number
	Number of hydrogen refueling stations	number
	Share of CO_2_ emissions from transport in total CO_2_ emissions	%
	Number of municipalities with developed sustainable mobility strategies	number
	Share of residents using public mass transport services	%
	Number of cars per household	number/household
	Carsharing – number of vehicles per 1000 citizens	number
ENERGY SECTOR	Share of energy from renewable sources in gross final energy consumption	%
	Electrical energy consumption	GWh
	The output of energy: Value added/energy consumption	TEUR/ktoe
	Total electricity consumption per Euro 1 million of GDP	GWh/1 MEUR
	Energy consumption per Euro 1 million of GDP	ktoe/1 MEUR
	Energy productivity (constant GDP to total primary energy consumption)	Euro/ktoe
	Share of biomass in energy production from RES	%
	Installed MW of biomass generation	MW
	Number of installations for agricultural biogas production	number
	Volume of biofuel production (bioethanol, biodiesel, biomethane)	m^3^
	Consumption of biofuels	ktoe
	The amount of by-products generated	Mg
	Share of managed by-products in total amount generated	%
	Life cycle assessment of enterprises activity (amount companies with LCA reports)	number
	Share of the sector in CO_2_ emissions	%
	Share of the sector in GHG emissions	%
SOCIO-INNOVATION AREA	Environment-related technologies patents	number
	Share of green jobs in total employment in regional companies	%
	Number of CE strategies/roadmaps developed by companies	number
	Number of CE strategies/roadmaps developed by municipalities	number
	Availability of facilities for repairing, reusing and sharing items	number
	Number of sharing economy projects	number
	Number of people participating in environmental education	number
	Number of information and education activities on CE	number
	Number of green public procurements	number
	Share of local GDP allocated to circular economy activities	%
	The amount of household food waste generated per capita	Mg

^*^Key industrial sectors: furniture, textiles, paper, machine and electromechanical; pkm – passenger-kilometers, MEUR – 1 million Euro, *TEUR* thousands Euro, *GHG* greenhouse gas, *GDP* gross domestic product, *EMAS* Eco-Management and Audit Scheme, *LCA* Life Cycle Assessment

## Discussion

EU members’ regional CE strategies highlight key sectors for their region. Many of them correspond to Wielkopolska’s sectors, i.e., agri-food [Emilia-Romagna, Andalusia, Madeira, Wallonia], industrial processing [Castilla-La Mancha, Tâmega e Sousa, Ostrobothnia], transport and mobility [Brussels-Capital Region], construction [Flanders, Wallonia], fuel and energy [Scotland, Extremadura]. Each region refers to aspects of the socio-innovation area. It should be noted that despite the selection of key sectors for the EU region from a CE perspective, not all EU strategies have matched indicators.

### Agri-food sector

The agri-food sector plays a prominent role in CE in terms of sustainable production (Klein et al. 2022). In some EU regions [i.a. Emilia-Romagna, Andalusia], the agri-food sector can be considered a strategic sector. It must meet the challenge of pursuing the production of healthy food in a sustainable manner and through efficient use of raw materials.

A major challenge of the agri-food sector is to minimize the amount of waste generated in the various links of the value chain [Castilla-La Mancha]. At the level of the first link – primary production, losses occur mainly due to adverse weather conditions, poor production practices, and failure to meet quality standards. On the other hand, in processing (the second link of the value chain), the problem of food waste arises, e.g., at the stage of storage of raw materials, their processing and warehousing of final products (KOWR 2021).

Among other possible ways to measure CE implementation is to analyze the mass of managed waste generated. Methods of treating organic waste produced in the agri-food sector include composting, fermentation, recovery of nutrients and processing into feed, as well as, in the case of inorganic waste, separate collection and recycling [Flanders]. The agri-food sector generates large amounts of plastic waste, which should be considered from the perspective of recyclability [Emilia-Romagna]. Not all plastic waste is recyclable due to its negligible or negative processing value (Li et al. 2023), therefore it should be replaced with other biodegradable materials [Galicia].

An important issue is the natural wastes, such as livestock by-products. They are accessible as a waste product from livestock farming, making them one of the locally available renewable energy resources (RES) such as biomass [Friesland]. Biomass is produced not only from animal, but also from plant waste. In Wielkopolska, about 10 Mg of plant biomass is harvested annually from 1 hectare of farmland, which is equivalent to about 5 tons of coal (Siudak et al. 2020). There is even greater potential for animal biomass resulting from developed livestock production. This potential is estimated at more than 1000 biogas plants, equivalent to 1 billion m^3^ of biomethane per year (Wdowin et al. 2021).

Organic residues can also be used to produce N and P fertilizers with wide application in soil improvement treatments. Increasing the circulation of N and P is being promoted to reduce the use of synthetic N and P fertilizers. At the same time, the EU has identified possible measures to minimize nutrient losses by 50% and reduce fertilizer use by at least 20% by 2030 (EC 2020b).

A significant issue for Wielkopolska is the energy intensity of the described sector. Despite a noticeable decline in solid fuel consumption, the food industry is still dependent on other fossil energy sources. The estimation of energy consumption varies greatly depending on the production processes used (Ladha-Sabur et al. 2019). In agriculture, on the other hand, energy intensity is influenced by the intensity of crop and livestock production and the agricultural production technologies applied (Wysokiński et al. 2017).

Other indicators include CO_2_ emissions and nitrogen compounds. Intensification of production has increased the role of the agri-food sector as an emitter of greenhouse gases. However, the impact of the agricultural system goes beyond greenhouse gas emissions [Flanders]. Emissions of ammonia (NH_3_) and nitrogen oxides (NO_x_) released from agriculture cause acidification and eutrophication of natural ecosystems, leading to ecosystem degradation (Liu et al. 2022).

In the agri-food sector, improvement is needed to organize the water management system. According to the new closed-loop paradigm, water is conceptualized as a basic resource to be managed as a scarce economic good with increasing value (Schoeman et al. 2014). Reducing water losses, recovering water from wastewater and promoting rainwater collection are among the ways to achieve this [Galicia, Madeira].

Soil is another vital resource. Its assessment can be accomplished by determining the percentage of land where soil quality, especially in terms of organic carbon, is maintained or improved concerning the total land (Velasco-Muñoz et al. 2021). On agricultural land, there should be a sustainable agricultural production system in which cultivation and soil management techniques protect the soil from erosion and degradation [Andalusia].

The agri-food sector should also emphasize pro-environmental aspects, which may include organic production, ecological certification and other initiatives. Organic farming combines environmental best practices, high levels of biodiversity, conservation and improvement of natural resources [Andalusia].

Various agro-ecological initiatives are proposed to promote ecological agriculture. Among them are workshops on regenerative agriculture, agro-ecological training, or reclaiming various urban spaces for agricultural projects [Extremadura].

### Construction sector

Another strategic sector for the regional CE is construction. It is estimated that this sector generates 100 billion tons of construction, renovation and demolition waste annually (UN 2022). The transition to waste-free construction requires multifaceted materials strategies and monitoring of change processes that consider the entire building life cycle and a systems approach (Diemer et al. 2022). This is particularly important due to the rapid growth of new buildings in Wielkopolska, including residential type by nearly 20% in 2021 (Statistics Poland 2022).

Life Cycle Assessment (LCA) allows analyzing a building holistically at all stages of the construction process (Wu et al. 2020). At the same time, the LCA methodology promotes a “cradle to cradle” approach. It is a method that covers the reuse, recovery, and recycle (3 R) potentials of the materials (Ng and Chau 2015). According to this methodology, the first step in construction should be circular design, which can positively influence innovation as a generator of symbiosis and synergy conducive to CE. Other steps include waste reduction, manageability and recyclability.

At the stage of demolition of buildings, all kinds of materials should be recycled to reduce the demand for primary raw materials [Scotland]. Construction practice should be geared toward achieving efficient use of resources. An important resource in this regard is soil. However, effective recovery of excavated soil faces obstacles in terms of logistics, costs or legislation.

In the construction sector, it is crucial to follow the path towards zero-emission [Wallonia]. New buildings in the EU must be zero-emission from 2030, and public ones from 2027 (EC 2021). To adapt the constructions to zero-carbon, it is advisable to maximize building insulation, minimize energy consumption and adopt RES to achieve energy self-sufficiency (Liu et al. 2016). In the EU regions, a gradual reduction in energy consumption can be observed, for example, through their thermal modernization.

One result of the green trend is environmental certifications. These indicate products and services with the best environmental value, which reduce the negative impact of buildings on the environment [Wallonia]. The most common certifications are Building Research Establishment Environmental Assessment Method – BREEAM, Leadership in Energy and Environmental Design – LEED, High Quality Environmental standard – HQE or Carbon Neutral Company (UN 2022).

### Mobility and transport sector

The transport and mobility sector also faces many challenges in meeting quality standards (EC 2019). This sector contributes significantly to environmental pollution mainly through energy consumption and high emissions (Huang et al. 2023). It requires extensive land area and thus contributes to urban sprawl and habitat fragmentation. Simultaneously, a significant number of new vehicle registrations of around 200,000 vehicles are observed annually in Wielkopolska (CEPiK 2022), which is closely related to the increase in the motorization rate.

The transition towards sustainable and integrated transport should include the management of public transport, alternative means of transport and e-mobility solutions (Scuttari and Isetti 2019). Implementing the principles of the CE model will allow the development of clean transport and mobility. Among the indicators of the sector are those related to the type of transport, the intensity of car use, the size of the mobility system, using public mass transport services, or the number of vehicle charging stations [i.a. Große Walsertal, Extremadura, Central Finland]. The ratio of cars per household was also considered relevant. In the EU in 2020, the average rate was 0.53 passenger cars per capita, while in Wielkopolska it was 0.72 (Eurostat 2023a). To increase the use of particular vehicles, an expansion of carsharing initiatives is proposed. Carsharing proves that mobility does not necessarily require individual ownership of vehicles (Kent and Dowling 2013) and maximizes environmental benefits [Flanders, Päijät-Häme].

Representatives of municipalities have an important role in promoting and encouraging investment in clean transportation modes and developing electromobility plans. Sustainable mobility strategies aim to accelerate the deployment of environmentally friendly vehicles and infrastructure, while recognizing the urgent need for comprehensive changes in transportation systems (TAPSEC 2023).

### Energy sector

Measures directed toward low-carbon solutions should particularly concern the energy sector. Today, municipalities consume close to two-thirds of the global energy, accounting for about 80% of the GHG emissions (Heshmati and Rashidghalam 2021). Their reduction is considered in the use of RES, e.g., biomass [i.a. Castilla-La Mancha, Catalonia, Andalusia]. In Wielkopolska, biomass is the most promising source of renewable energy. It is estimated that about 10 tons of biomass are collected annually from 1 hectare of agricultural land, which is equivalent to about 5 tons of coal (Siudak et al. 2020). In addition, there is a very high potential in Wielkopolska for the development of the market for agricultural biogas plants. Currently, the region has the largest agricultural biogas installation in Poland and the largest biomass units at the Konin Power Plant (ZEPAK 2022). Meanwhile, Wielkopolska ranks only in the middle of the rate compared to the other Polish provinces in terms of the share of RES in electricity production (34%) (Statistics Poland 2022).

EU regional strategies also pay attention to biofuel production (bioethanol, biodiesel, biomethane) [Andalusia]. In Wielkopolska, bioethanol is produced by 6 installations. Soon biomethane will be generated in the first biogas plant producing this biofuel (Ministry of Agriculture and Rural Development 2022). The use of RES, including biofuels, will positively affect the reduction of GHG emissions, including CO_2_. Emissions in the energy systems sector are primarily from electricity and heat generation in power plants (e.g., coal and gas power stations), as well as the extraction, processing and transport of fuels for these plants (Lamb et al. 2022).

Reducing energy consumption also represents a significant potential for reducing CO_2_ emissions. Energy consumption per unit of value added is one way of measuring energy requirements and energy efficiency. Relating energy consumption to GDP indicates the general relationship of energy consumption to economic growth (Fuinhas and Marques 2012).

The energy sector is also a source of by-products that result from using fossil fuels. According to CE, these wastes should be managed in other industries to reduce the amount of unmanaged final waste [Galicia]. Support in closing the loop are environmental management systems which propose the use of a life cycle assessment of enterprises activity (Smol et al. 2017).

### Industrial processing sector

As in the energy sector, the industrial processing is distinguished by instruments aimed at supporting pro-environmental activities. These are tools in the field of environmental management (Eco-Management and Audit Scheme - EMAS), reporting of company performance (LCA report) (Smol et al. 2017), and product certification (Ecolabel) (Courtat et al. 2023). In addition, innovative projects in the field of industrial and territorial ecology are of great importance (Aranda-Usón et al. 2020).

The next groups of indicators are in line with CE’s environmental goals of striving to minimize waste generation, water and energy consumption. They are related to key industries for Wielkopolska, which are reflected in EU regional strategies: board and furniture manufacturing [Tâmega e Sousa, Castilla-La Mancha], textiles [Wallonia, Andalusia], paper [Andalusia], and machine and electromechanical industry [Emilia-Romagna, Catalonia]. The most environmentally burdensome industries would implement recovery measures [Extremadura].

Another CE indicator analyzed in the industrial processing sector is an industrial value added, which fits into the economic objective of CE (Eurostat 2023b). It helps determine which sector contributes the most to the production of total goods and services and its importance for the region. It is necessary to develop and position these sectors in the market in the context of CE to create circular products, services, and green jobs. Especially while industrial processing represents the highest share of employment in the Wielkopolska region (37.3%) (Marshal’s Office of the Wielkopolskie Voivodeship 2022).

Referring to the analyzed industries and their impact on the environment, the textile industry is considered the most polluting industry (Kazancoglu et al. 2020). All production processes in this business are energy- and water-intensive and consume excessive resources. The pulp and paper industry using wood raw materials also stands out in terms of the large amount of waste created (Dixit et al. 2020). The production process generates solid wood waste, but also toxic emissions and wastewater. It is an energy- and water-intensive industry (Kong et al. 2016).

On the other hand, the machine and electromechanical industry is a good example of reusing large amounts of recovered raw materials, e.g., ferrous and non-ferrous metals, plastics, rare metals and many other materials. However, the complete e-waste recycling process is still underdeveloped (Abdelbasir et al. 2018; Ahirwar and Tripathi 2021). In terms of disposal, a notable problem is a special waste, e.g., industrial liquids, cooling fluids, and cutting oils, which require additional processing, special transport and packaging, and often non-standard landfill techniques.

### Socio-innovation area

The last group of indicators is represented by the socio-innovation area. At the regional level, an increase in the number of green public procurement (GPP) is proposed, which can be a major driver for CE innovation [Scotland]. In addition, their application can shape production and consumption trends, while significant demand from public institutions for “greener” goods will create or expand markets for environmentally friendly products and services (EC 2008).

CE implementations can also be described by the number of patents in environment-related technologies (Silvestri et al. 2020) [Aragón, Castilla-La Mancha]. This information is important to measure the results of innovation policies. Indirectly, the implementation of patented inventions leads to the development of the region by increasing the use of cleaner technologies (OECD 2023).

A multidimensional indicator of CE development is the growth of green jobs (Avdiushchenko and Zając 2019). CE-related jobs can contribute to the generation of environmentally beneficial goods or services, such as green buildings or clean transport. At the same time, these jobs can be ecological if they use more ecosystem-friendly processes, improving energy and raw material efficiency, reducing greenhouse gas emissions, or minimizing waste and pollution.

Developing strategies or roadmaps for a closed-loop economy both at the level of individual economic entities or authorities is also an important issue. From a social or economic point of view, these should identify model solutions for extending the lifetime of goods through the sharing economy [i.a. Chemport, Wallonia, North Karelia], reparation and reuse [i.a. Flanders, Catalonia], e.g., waste electrical and electronic equipment, which has become the fastest growing waste stream in the world (Ylä-Mella and Román 2019).

From an economic point of view, among social and innovation indicators, a local GDP allocated to CE activities is proposed [Brussels*-*Capital Region]. In terms of this indicator, it is emphasized that the CE model affects economic growth (Grdic et al. 2020; Lacko et al. 2021).

Many EU regional strategies also highlight environmental attitudes and values as well as the dissemination of green knowledge and education activities [i.a. Chemport, Flanders], e.g., environmental competitions, eco-campaigns promoting waste management hierarchy (Avdiushchenko and Zając 2019). Among educational campaigns, countering food waste is often promoted [i.a. Extremadura, Galicia]. Along the food supply chain, households generate the largest share of food waste (Schanes et al. 2018).

## Conclusions

The transition to the CE model requires the establishment of recommendations for comprehensive support of this process, including monitoring of its implementation. The guidelines should be consistent with the region’s specifics, considering that the heterogeneities between provinces are enormous. Although different regions of EU member states use a consistent conceptual basis for CE strategies, the creation of regional roadmaps, including indicators, requires taking into account the specific context of each region. There is no consensus on the best way to track the various CE activities.

The article contributes to the selection of appropriate indicators for the targeted region while encouraging other provinces to develop individually adapted, holistic systems for monitoring CE implementation. The success of policies supporting a green, closed-loop economy will largely depend on the actual integration of the CE model across consolidated economic sectors, and therefore stakeholder engagement. The indicators presented can be used in regional policy-making, including as a decision-making instrument to support CE implementation. Understanding their relevance may be a vital for determining priorities for development strategies, funding programs and research projects. They can also provide a basis for final indicators in regional CE policy.

However, this study is not without limitations. First, this paper examines only regional strategy from European Circular Economy Stakeholder Platform. Further analysis could be enriched to include strategies from other EU regions, as well as consider other source bases. Also, the literature review included only academic journal articles. Future studies could also include gray literature and differentiate between business and academic approaches. Furthermore, due to the number of indicators, it is worthwhile to further develop a tool for aggregating indicators and setting thresholds with possible forecasting of the meeting of target values in the region. Additionally, the availability of the indicated statistics at the regional level remains the exception rather than the norm. Stakeholders should be engaged to collect the necessary data to track progress toward CE at the indicated level of detail.

Furthermore, it is essential to point out the need for a study of each region in the future, as necessary for the further development of consistent indicators for the country based on developed consolidated regional indications, rather than just top-down governmental indications. In this context, the monitoring framework presented in this work may represent the first step toward a consolidated and harmonized system based on subnational territories.

### Supplementary information

Supplementary Information
