# Supplementary material for: Indicators for a circular economy in a regional context: an approach based on Wielkopolska region, Poland
Source: Environ Manage. 2023 Sep 26;73(2):293–310. doi: 10.1007/s00267-023-01887-w (PMC10847184; doi:10.1007/s00267-023-01887-w)
Supplement: Supplementary file 1 — Supplementary Information [file 267_2023_1887_MOESM1_ESM.pdf]

Table A Selected indicators by sector and area for Wielkopolska province with consideration of regional strategies and scientific literature.

Sector/ area	Indicators	Unit	Connection to the strategy[1]	Support in the literature[2]
AGRI-FOOD SECTOR	The amount of food waste	Mg	Große Walsertal, Castilla-La Mancha, Galicia, Extremadura, Scotland	Heshmati and Rashidghalam, 2021
	Share of food waste from the sector to total food waste	%	Madeira, Castilla-La Mancha, Extremadura	-
	Share of managed waste generated in the sector to total waste	%	Große Walsertal, Andalusia, Castilla-La Mancha, Galicia	Wang et al., 2015
	Plastic usage	Mg	Emilia-Romagna, Galicia, Castilla-La Mancha	-
	The amount of managed livestock by-products	Mg	Friesland	Papangelou and Mathijs, 2021
	The amount of biomass product per unit area	Mg/ha	Andalusia, Extremadura, Galicia	Hildebrandt et al., 2020
	Total consumption of synthetic N and P fertilizers in agricultural production per unit area	Mg/ha	Andalusia, Castilla-La Mancha, Extremadura, Flanders, Friesland	Guo-gang, 2011; Papangelou and Mathijs, 2021; Wang et al., 2015
	Total consumption of organic N and P fertilizers in agricultural production per unit area	Mg/ha	Päijät-Häme, Castilla-La Mancha, Galicia	Wang et al., 2015
	Percentage of land with maintained or improved soil quality relative to total land	%	Extremadura, Große Walsertal	-
	CO_2 emissions in relation to the CO_2 total emissions	%	Große Walsertal	-
	Nitrogen compound emissions (NH_3, NO_x) to the nitrogen compound total emissions	%	Flanders	-
	Water consumption	m^3	Extremadura	-
	Share of the agri-food sector in total water consumption	%	Flanders	-
	Share of reused water in total water use	%	Andalusia, Castilla-La Mancha, Flanders	Papangelou and Mathijs, 2021
	The amount of rainwater used	m^3	selected only on the basis of expert recommendations	
	Number of producers with organic certification	number	Madeira, Andalusia	Hildebrandt et al., 2020
	Number of organic farms per unit area of agricultural land	number/ 1,000 ha	Extremadura	Muizniece et al., 2019
	Percentage of the cultivated area under organic production	%	Päijät-Häme	-

Sector/ area	Indicators	Unit	Connection to the strategy[1]	Support in the literature[2]
	Energy consumption of the sector in relation to total demand in the region	%	Andalusia	-
	Number of agro-ecological initiatives	number	Extremadura, Andalusia	-
CONSTRUCTION SECTOR	Share of construction and demolition waste in general waste	%	Flanders	-
	The amount of construction and demolition waste generated	Mg	Flanders, Brussels-Capital Region	-
	Share of managed construction and demolition waste in the total amount of waste	%	Flanders, Scotland, Galicia, Madeira	Mihai, 2019; Wang et al., 2018
	Recycling rate of construction and demolition waste	%	Ostrobothnia, Central Finland, North Karelia, South Karelia, Southwest Finland, Flanders, Große Walsertal, Scotland	-
	Products and construction techniques covered by life cycle analysis studies	number	Flanders	-
	Construction works with circular design	%	Scotland, Galicia	-
	Share of reused excavated soil in the total amount of construction and demolition waste	%	Päijät-Häme, Flanders	-
	Share of new zero-emission buildings in the total number of new buildings	%	Friesland, Catalonia	Wang et al., 2018
	Share of RES in total energy consumption in public buildings	%	selected only on the basis of expert recommendations	
	Share of public buildings requiring thermal modernization	%	selected only on the basis of expert recommendations	
	Construction works with a minimum level of materials reuse (%)	%	Flanders, Galicia	-
	Share of buildings with certification in relation to all buildings	%	Wallonia	-
INDUSTRIAL PROCESSING SECTOR	Industrial value added in the key sectors*	Euro	Flanders, Extremadura	Su et al., 2013
	Total amount of generated industrial waste	Mg	Castilla-La Mancha, Flanders	Arbolino et al., 2020; Schilkowski et al., 2020; Smol et al., 2017
	Industrial waste generation per unit of industrial value added	Mg/Euro	-	Arbolino et al., 2020; Guo et al., 2017
	Industrial waste generation in the key industrial sectors*	Mg	-	Arbolino et al., 2020; Guo et al., 2017
	Recycling rate of industrial solid waste	%	Emilia-Romagna	Arbolino et al., 2020; Geng et al., 2009; Guo-gang, 2011; Hildebrandt et al., 2020; Jia and Zhang, 2011; Silvestri et al., 2020; Smol et al., 2017; Su et al.,

Sector/ area	Indicators	Unit	Connection to the strategy[1]	Support in the literature[2]
				2013; Towa et al., 2021; Wang et al., 2015
	Industrial waste produced as % of the total waste produced	%	-	Arbolino et al., 2020; Schilkowski et al., 2020; Smol et al., 2017
	Industrial waste reused as a source of raw materials in relation to total waste	%	Galicia, Wallonia	Arbolino et al., 2020; Guo et al., 2017
	CO_2 emissions resulting from production processes	Mg	Tâmega e Sousa	Arbolino et al., 2020
	SO_2 emissions resulting from production processes	Mg	-	Geng et al., 2009; Guo et al., 2017; Hu et al., 2018
	Share of the sector in GHG emissions	%	Tâmega e Sousa, Emilia-Romagna, Castilla, Catalonia, Galicia	Gao et al., 2020
	Energy consumption per unit of industrial value added	ktoe/ TEUR	-	Arbolino et al., 2020; Geng et al., 2012; Guo et al., 2017; Su et al., 2013
	Energy consumption per unit of production in the key industrial sectors*	ktoe/ TEUR	-	Geng et al., 2012; Hu et al., 2018; Su et al., 2013
	Energy consumption of the key industrial sectors in the total regional energy consumption	%	-	Geng et al., 2012; Su et al., 2013
	Water intensity of industry - industrial water consumption per unit of industrial value added	m^3/TEUR	-	Arbolino et al., 2020; Geng et al., 2009; Guo et al., 2017; Su et al., 2013
	Water consumption per unit of production in the key industrial sectors*	m^3/TEUR	-	Geng et al., 2012; Hu et al., 2018; Su et al., 2013
	Industrial wastewater generation per unit of industrial value added	m^3/TEUR	-	Su et al., 2013
	Reuse rate of industrial water	%	Große Walsertal, Wallonia	Guo et al., 2017; Jia and Zhang, 2011; Ning, 2012; Smol et al., 2020; Su et al., 2013
	Life cycle assessment of enterprises activity (amount companies with LCA reports)	number	-	Husgafvel et al., 2017; Smol et al., 2017
	Number of EMAS implementations in entities	number	Castilla-La Mancha, Catalonia, Galicia	-

Sector/ area	Indicators	Unit	Connection to the strategy[1]	Support in the literature[2]
	Number of Ecolabel-certified products/services	numbers	Galicia, Tâmega e Sousa	Aranda-Usón et al., 2020; Avdiushchenko, 2018
	Industrial and territorial ecology projects	numbers	selected only on the basis of expert recommendations	
MOBILITY AND TRANSPORT SECTOR	Passenger transport - total movement of passengers using public transport	pkm	Flanders	Alaerts et al., 2019
	Share of petrol-powered vehicles to total registered vehicles, with their number for:	%	Flanders	Alaerts et al., 2019
	- passenger vehicles	number		
	- mass passenger transport vehicles	number		
	- freight transport vehicles	number		
	Share of diesel-powered vehicles to total registered vehicles, with their number for:	%	Flanders	Alaerts et al., 2019
	- passenger vehicles	number		
	- mass passenger transport vehicles	number		
	- freight transport vehicles	number		
	Share of electric vehicles to total registered vehicles, with their number for:	%	Catalonia, Päijät-Häme	Alaerts et al., 2019; Heshmati and Rashidghalam, 2021
	- passenger vehicles	number		
	- mass passenger transport vehicles	number		
	- freight transport vehicles	number		
	Share of hydrogen vehicles to total registered vehicles, with their number for:	%	-	Alaerts et al., 2019; Heshmati and Rashidghalam, 2021
	- passenger vehicles	number		
	- mass passenger transport vehicles	number		
	- freight transport vehicles	number		
	Environmental cars in the municipal organization	number	Tâmega e Sousa, Große Walsertal	Heshmati and Rashidghalam, 2021

Sector/ area	Indicators	Unit	Connection to the strategy[1]	Support in the literature[2]
	Number of stations and charging points for electric vehicles	number	Päijät-Häme, Extremadura, Große Walsertal, Ostrobothnia, Central Finland, North Karelia, South Karelia, Southwest Finlandia	-
	Number of hydrogen refueling stations	number	selected only on the basis of expert recommendations	
	Share of CO_2 emissions from transport in total CO_2 emissions	%	Päijät-Häme	-
	Number of municipalities with developed sustainable mobility strategies	number	Große Walsertal	-
	Share of residents using public mass transport services	%	Flanders	Avdiushchenko and Zając, 2019
	Number of cars per household	number/ household	Flanders	-
	Carsharing - number of vehicles per 1000 citizens	number	Große Walsertal, Flanders, Galicia, Päijät-Häme	-
ENERGY SECTOR	Share of energy from renewable sources in gross final energy consumption	%	Extremadura, Andalusia, Brussels-Capital Region, Friesland, Päijät-Häme, Tâmega e Sousa, Große Walsertal	Avdiushchenko, 2018; Avdiushchenko and Zając, 2019; Heshmati and Rashidghalam, 2021; Hildebrandt et al., 2020
	Electrical energy consumption	GWh	Tâmega e Sousa	-
	The output of energy: Value added/energy consumption	TEUR/ ktoe	Madeira	Geng et al., 2012
	Electricity consumption per Euro 1 million of GDP	GWh/ MEUR	Madeira	Arbolino et al., 2020; Avdiushchenko and Zając, 2019; Geng et al., 2012; Guo et al., 2017; Qing et al., 2011; Su et al., 2013; Tang et al., 2020
	Energy consumption per Euro 1 million of GDP	ktoe/ MEUR	Extremadura	(Geng et al., 2009; Guo et al., 2017; Guo-gang, 2011; Hu et al., 2018; Ning, 2012; Wang et al., 2015)
	Energy productivity (constant GDP to total primary energy consumption)	Euro/ktoe	-	Arbolino et al., 2020; Avdiushchenko, 2018; Geng et al., 2012; Hildebrandt et al., 2020; Jia and Zhang, 2011; Tang et al., 2020
	Share of biomass in energy production from RES	%	Castilla	-

Sector/ area	Indicators	Unit	Connection to the strategy[1]	Support in the literature[2]
	Installed MW of biomass generation	MW	Andalusia, Extremadura	-
	Number of installations for agricultural biogas production	number	Andalusia, Extremadura, Galicia	-
	Volume of biofuel production (bioethanol, biodiesel, biomethane)	m^3	Andalusia	Avdiushchenko and Zając, 2019; Heshmati and Rashidghalam, 2021
	Consumption of biofuels	ktoe	Andalusia, Extremadura	Guo et al., 2017
	The amount of by-products generated	Mg	Andalusia, Catalonia, Extremadura, Galicia, Scotland	-
	Share of managed by-products in total amount generated	%	Andalusia, Catalonia, Galicia, Scotland, Wallonia	-
	Life cycle assessment of enterprises activity (amount companies with LCA reports)	number	-	Smol et al., 2017
	Share of the sector in CO_2 emissions	%	Flanders, Tâmega e Sousa	-
	Share of the sector in GHG emissions	%	Extremadura, Madera, Tâmega e Sousa, Andalusia	Avdiushchenko, 2018; Heshmati and Rashidghalam, 2021; Hildebrandt et al., 2020; Tang et al., 2020; Wang et al., 2015
**SOCIO-INNOVATION AREA**	Environment-related technologies patents	number	Madeira, Tâmega e Sousa, Aragón	Avdiushchenko, 2018; Avdiushchenko and Zając, 2019; Silvestri et al., 2020
	Share of green jobs in total employment in regional companies	%	Brussels-Capital Region, Wallonia, Scotland, Galicia, Aragón	Aranda-Usón et al., 2020; D'Adamo et al., 2022; Hildebrandt et al., 2020; Scarpellini et al., 2019; Smol et al., 2017
	Number of CE strategies/roadmaps developed by companies	number	-	Alonso-Almeida and Rodríguez-Antón, 2020; Aranda-Usón et al., 2020; Avdiushchenko, 2018; Smol et al., 2017
	Number of CE strategies/roadmaps developed by municipalities	number	-	
	Availability of facilities for repairing, reusing and sharing items	number	Große Walsertal, Wallonia, Tâmega e Sousa, Scotland, Päijät-Häme, Galicia, Flanders, Extremadura, Chemport, Catalonia, Castilla-La Mancha, Brussels-Capital Region, Andalusia	Avdiushchenko, 2018; Avdiushchenko and Zając, 2019; Heshmati and Rashidghalam, 2021; Wang et al., 2018

Sector/ area	Indicators	Unit	Connection to the strategy[1]	Support in the literature[2]
	Number of sharing economy projects	number	Große Walsertal, Amsterdam, Chemport, Emilia-Romagna, Flanders, Päijät-Häme, Wallonia, Ostrobothnia, Central Finland, North Karelia, South Karelia, Southwest Finland	Alonso-Almeida and Rodríguez-Antón, 2020; Avdiushchenko, 2018; Wang et al., 2018
	Number of people participating in environmental education	number	Brussels-Capital Region, Aragón, Ostrobothnia, Central Finland, North Karelia, South Karelia, Southwest Finlandia	Avdiushchenko and Zając, 2019; Wang et al., 2015
	Number of information and education activities on CE	number	Galicia, Flanders, Extremadura, Chemport, Castilla-La Mancha, Andalusia, Große Walsertal	Alonso-Almeida and Rodríguez-Antón, 2020; Avdiushchenko, 2018; Wang et al., 2018, 2015
	Number of green public procurements	number	Große Walsertal, Wallonia, Tâmega e Sousa, Scotland, Päijät-Häme	Avdiushchenko, 2018; Avdiushchenko and Zając, 2019; Wang et al., 2018
	Share of local GDP allocated to circular economy activities	%	Brussels-Capital Region	-
	The amount of household food waste generated per capita	Mg	Galicia, Scotland, Castilla-La Mancha, Extremadura, Flanders	-

*key industrial sectors: furniture, textiles, paper, machine and electromechanical; pkm - passenger-kilometres, MEUR - 1 million Euro, TEUR - thousands Euro; GHG – greenhouse gas, GDP – gross domestic product, EMAS – Eco-Management and Audit Scheme, LCA – Life Cycle Assessment

1 - The details of the strategies are summarized in the following Table B; 2 – The details are included in the References

Table B Reference data on CE regional strategies.

No.	Country	Region	Year of publication	References
1	Austria	Große Walsertal (covers Vorarlberg region)	2020	Regio Großes Walsertal, 2020. A circular economy strategy for the Großes Walsertal [WWW Document]. URL www.grosseswalsertal.at (accessed 1.30.23).
2	Belgium	Brussels-Capital Region	2016	Government of the Brussels-Capital Region, 2016. Regional Program for a Circular Economy, Brussels Capital Region [WWW Document]. URL https://document.environnement.brussels/opac_css/elecfile/PROG_160308_PREC_DEF_FR (accessed 1.29.23).
3	Belgium	Flanders	2020	Circular Economy Policy Research Center, 2020. Overview Circular Flanders: a 2017-2019 retrospective [WWW Document]. URL https://www.vlaanderen-circulair.be/en/retrospect (accessed 1.29.23).
4	Belgium	Wallonia	2021	Walloon Public Service, 2021. Circular Wallonia [WWW Document]. URL https://economiecirculaire.wallonie.be/ (accessed 1.29.23).
5	Finland	Ostrobothnia	2020	Jakobstad Region Development Company Concordia, 2020. Ostrobothnia in transition - the Roadmap for Sustainable Development and Circular Economy [WWW Document]. URL https://www.jakobstadsregionen.fi/wp-content/uploads/2021/10/Roadmap.pdf (accessed 1.29.23).
6	Finland	Central Finland	2018	Circwaste, 2018a. Central Finland’s circular economy roadmap [WWW Document]. URL https://www.materiaalitkiertoon.fi/fi-FI/Tyokalut/Kiertotalouden_tiekartat/Alueiden_tiekartat (accessed 1.29.23).
7	Finland	North Karelia	2018	Circwaste, 2018b. North Karelia’s circular economy roadmap [WWW Document]. URL https://www.materiaalitkiertoon.fi/fi-FI/Tyokalut/Kiertotalouden_tiekartat/Alueiden_tiekartat (accessed 1.29.23).
8	Finland	South Karelia	2018	Circwaste, 2018c. South Karelia’s circular economy roadmap [WWW Document]. URL https://www.materiaalitkiertoon.fi/fi-FI/Tyokalut/Kiertotalouden_tiekartat/Alueiden_tiekartat (accessed 4.29.23).
9	Finland	Southwest Finland	2019	Circwaste - Materiaalit kiertoon, 2019. Southwest Finland’s circular economy roadmap [WWW Document]. URL https://www.materiaalitkiertoon.fi/fi-FI/Tyokalut/Kiertotalouden_tiekartat/Alueiden_tiekartat (accessed 2.21.23).
10	Finland	Päijät-Häme	2021	The Regional Council of Päijät-Häme, 2021. Päijät-Häme’s circular strategy [WWW Document]. URL http://www.kohtikiertotaloutta.fi/english/ (accessed 1.30.23).
11	Italy	Emilia-Romagna	2019	Regione Emilia-Romagna, 2019. Regional strategy for reducing the impact of plastics on the environment - #Plastic-freER [WWW Document]. URL https://ambiente.regione.emilia-romagna.it/it/rifiuti/temi/rifiuti/economia-circolare/strategia-plasticfreer-1 (accessed 1.30.23).

No.	Country	Region	Year of publication	References
12	Netherlands	Chemport (covers: the Province of Groningen, the Province of Drenthe)	2020	Chemport Europe, 2020. A saccharide strategy for the Dutch Chemport Region [WWW Document]. URL https://www.chemport.eu/downloads/ (accessed 1.29.23).
13	Netherlands	Friesland	2021	Circulair Friesland, 2021. Circular Friesland's three lines of action: Doing. Learning. Telling. [WWW Document]. URL https://circulairfriesland.frl/en/ (accessed 1.29.23).
14	Portugal	Madeira	2021	Portuguese Regional Directorate for the Environment and Climate Change, 2021. Madeira Circular Agenda [WWW Document]. URL https://www.madeira.gov.pt//Portals/12/Documentos/Noticias/AREC_Relat%c3%b3rio%20Final.pdf (accessed 1.30.23).
15	Portugal	Tâmega e Sousa	2019	Intermunicipal Community of Tâmega and Sousa, 2019. Tâmega e Sousa guide for local public authorities to promote circular economy [WWW Document]. URL https://www.cimtamegaesousa.pt/#/documentacao (accessed 1.29.23).
16	Spain	Andalusia	2018	Ministry of Agriculture Fisheries and Rural Development, 2018. The Andalusian circular bioeconomy strategy [WWW Document]. URL https://www.juntadeandalucia.es/organismos/transparencia/planificacion-evaluacion-estadistica/planes/detalle/155202.html (accessed 1.30.23).
17	Spain	Aragón	2020	Government of Aragón, 2020. Aragón Circular Strategy [WWW Document]. URL https://aragoncircular.es/ (accessed 1.29.23).
18	Spain	Castilla-La Mancha	2021	Dirección General de Economía Circular CLM, 2021. Castilla-La Mancha's Circular Economy Strategy for 2030 [WWW Document]. URL https://www.castillalamancha.es/gobierno/desarrollosostenible/estructura/dgecocir/actuaciones/estrategia-de-econom%C3%ADa-circular-2030-2021-2030 (accessed 1.29.23).
19	Spain	Catalonia	2015	Government of Catalonia, 2015. Strategy of the Government of Catalonia: Promoting Green and Circular Economy in Catalonia [WWW Document]. URL https://mediambient.gencat.cat/ca/05_ambits_dactuacio/empresa_i_produccio_sostenible/economia_verda/impuls_economia_verda/ (accessed 1.29.23).
20	Spain	Extremadura	2017	Regional Government of Extremadura, 2017. Extremadura 2030 [WWW Document]. URL https://extremadura2030.com/wp-content/uploads/2018/05/estrategia2030.pdf (accessed 1.30.23).
21	Spain	Galicia	2021	Xunta de Galicia, 2021. Galician circular economy strategy. Xacobeo 1–181.

No.	Country	Region	Year of publication	References
22	UK	Scotland	2016	The Scottish Government, 2016. Making Things Last: A Circular Economy Strategy for Scotland [WWW Document]. URL https://www.gov.scot/publications/making-things-last-circular-economy-strategy-scotland/documents/ (accessed 1.30.23).

References

Alaerts, L., Van Acker, K., Rousseau, S., De Jaeger, S., Moraga, G., Dewulf, J., De Meester, S., Van Passel, S., Compernolle, T., Bachus, K., Vrancken, K., Eyckmans, J., 2019. Towards a more direct policy feedback in circular economy monitoring via a societal needs perspective. Resour Conserv Recycl 149, 363–371. https://doi.org/10.1016/j.resconrec.2019.06.004

Alonso-Almeida, M.D.M., Rodríguez-Antón, J.M., 2020. The role of institutional engagement at the macro level in pushing the circular economy in Spain and its regions. Int J Environ Res Public Health 17. https://doi.org/10.3390/ijerph17062086

Aranda-Usón, A., Portillo-Tarragona, P., Scarpellini, S., Llena-Macarulla, F., 2020. The progressive adoption of a circular economy by businesses for cleaner production: An approach from a regional study in Spain. J Clean Prod 247. https://doi.org/10.1016/j.jclepro.2019.119648

Arbolino, R., Boffardi, R., Ioppolo, G., 2020. An insight into the Italian chemical sector: How to make it green and efficient. J Clean Prod 264. https://doi.org/10.1016/j.jclepro.2020.121674

Avdiushchenko, A., 2018. Toward a circular economy regional monitoring framework for European regions: Conceptual approach. Sustainability 10. https://doi.org/10.3390/su10124398

Avdiushchenko, A., Zając, P., 2019. Circular economy indicators as a supporting tool for european regional development policies. Sustainability 11, 3025. https://doi.org/10.3390/su11113025

D'Adamo, I., Falcone, P.M., Imbert, E., Morone, P., 2022. Exploring regional transitions to the bioeconomy using a socio-economic indicator: the case of Italy. Economia Politica 39, 989–1021. https://doi.org/10.1007/s40888-020-00206-4

Gao, Chengkang, Gao, Chengbo, Song, K., Fang, K., 2020. Pathways towards regional circular economy evaluated using material flow analysis and system dynamics. Resour Conserv Recycl 154. https://doi.org/10.1016/j.resconrec.2019.104527

Geng, Y., Fu, J., Sarkis, J., Xue, B., 2012. Towards a national circular economy indicator system in China: An evaluation and critical analysis. J Clean Prod 23, 216–224. https://doi.org/10.1016/j.jclepro.2011.07.005

Geng, Y., Zhu, Q., Doberstein, B., Fujita, T., 2009. Implementing China's circular economy concept at the regional level: A review of progress in Dalian, China. Waste Management 29, 996–1002. https://doi.org/10.1016/j.wasman.2008.06.036

Guo, B., Geng, Y., Ren, J., Zhu, L., Liu, Y., Sterr, T., 2017. Comparative assessment of circular economy development in China's four megacities: The case of Beijing, Chongqing, Shanghai and Urumqi. J Clean Prod 162, 234–246. https://doi.org/10.1016/j.jclepro.2017.06.061

Guo-gang, J., 2011. Empirical Analysis of Regional Circular Economy Development--Study Based on Jiangsu, Heilongjiang, Qinghai Province. Energy Procedia 5, 125–129. https://doi.org/https://doi.org/10.1016/j.egypro.2011.03.023

Heshmati, A., Rashidghalam, M., 2021. Assessment of the urban circular economy in Sweden. J Clean Prod 310. https://doi.org/10.1016/j.jclepro.2021.127475

Hildebrandt, J., Bezama, A., Thraen, D., 2020. Insights from the Sustainability Monitoring Tool SUMINISTRO Applied to a Case Study System of Prospective Wood-Based Industry Networks in Central Germany. Sustainability 12. https://doi.org/10.3390/su12093896

Hu, Y., He, X., Poustie, M., 2018. Can legislation promote a circular economy? A material flow-based evaluation of the circular degree of the Chinese economy. Sustainability 10. https://doi.org/10.3390/su10040990

Husgafvel, R., Poikela, K., Honkatukia, J., Dahl, O., 2017. Development and piloting of sustainability assessment metrics for arctic process industry in Finland-The biorefinery investment and slag processing service cases. Sustainability 9. https://doi.org/10.3390/su9101693

Jia, C.R., Zhang, J., 2011. Evaluation of regional circular economy based on matter element analysis, in: Procedia Environmental Sciences. Elsevier B.V., pp. 637–642. https://doi.org/10.1016/j.proenv.2011.12.099

Mihai, F.C., 2019. Construction and demolition waste in Romania: The route from illegal dumping to building materials. Sustainability 11. https://doi.org/10.3390/su11113179

Muizniece, I., Zihare, L., Pubule, J., Blumberga, D., 2019. Circular Economy and Bioeconomy Interaction Development as Future for Rural Regions. Case Study of Aizkraukle Region in Latvia. Environmental and Climate Technologies 23, 129–146. https://doi.org/10.2478/rtuect-2019-0084

Ning, L., 2012. Spatial variation and space optimization of the development of china’s circular economy. Chinese Journal of Population Resources and Environment 10, 51–59. https://doi.org/10.1080/10042857.2012.10685076

Papangelou, A., Mathijs, E., 2021. Assessing agro-food system circularity using nutrient flows and budgets. J Environ Manage 288. https://doi.org/10.1016/j.jenvman.2021.112383

Qing, Y., Qiongqiong, G., Mingyue, C., 2011. Study and integrative evaluation on the development of circular economy of Shaanxi Province, in: Energy Procedia. Elsevier Ltd, pp. 1568–1578. https://doi.org/10.1016/j.egypro.2011.03.268

Scarpellini, S., Portillo-Tarragona, P., Aranda-Usón, A., Llena-Macarulla, F., 2019. Definition and measurement of the circular economy’s regional impact. Journal of Environmental Planning and Management 62, 2211–2237. https://doi.org/10.1080/09640568.2018.1537974

Schilkowski, C., Shukla, M., Choudhary, S., 2020. Quantifying the circularity of regional industrial waste across multi-channel enterprises. Ann Oper Res 290, 385–408. https://doi.org/10.1007/s10479-019-03168-4

Silvestri, F., Spigarelli, F., Tassinari, M., 2020. Regional development of Circular Economy in the European Union: A multidimensional analysis. J Clean Prod 255. https://doi.org/10.1016/j.jclepro.2020.120218

Smol, M., Adam, C., Preisner, M., 2020. Circular economy model framework in the European water and wastewater sector. J Mater Cycles Waste Manag 22, 682–697. https://doi.org/10.1007/s10163-019-00960-z

Smol, M., Kulczycka, J., Avdiushchenko, A., 2017. Circular economy indicators in relation to eco-innovation in European regions. Clean Technol Environ Policy 19, 669–678. https://doi.org/10.1007/s10098-016-1323-8

Su, B., Heshmati, A., Geng, Y., Yu, X., 2013. A review of the circular economy in China: Moving from rhetoric to implementation. J Clean Prod 42, 215–227. https://doi.org/10.1016/j.jclepro.2012.11.020

Tang, J., Tong, M., Sun, Y., Du, J., Liu, N., 2020. A spatio-temporal perspective of China's industrial circular economy development. Science of the Total Environment 706. https://doi.org/10.1016/j.scitotenv.2019.135754

Towa, E., Zeller, V., Achten, W.M.J., 2021. Assessing the circularity of regions: Stakes of trade of waste for treatment. J Ind Ecol 25, 834–847. https://doi.org/10.1111/jiec.13106

Wang, N., Lee, J.C.K., Zhang, J., Chen, H., Li, H., 2018. Evaluation of Urban circular economy development: An empirical research of 40 cities in China. J Clean Prod 180, 876–887. https://doi.org/10.1016/j.jclepro.2018.01.089

Wang, Y., Sun, M., Wang, R., Lou, F., 2015. Promoting regional sustainability by eco-province construction in China: A critical assessment. Ecol Indic 51, 127–138. https://doi.org/10.1016/j.ecolind.2014.07.003
